# The Ward Laboratory.—I

**Published:** 1912-05-04

**Authors:** 


### THE MAKING OF A MODERN HOSPITAL.*

### The Ward Laboratory.—I.

Formerly every hospital ward contained a cupboard, a window-sill, or a special table which was styled the "examination cupboard," "test place," or "urine table," as the case might be. At this specially set apart working place the medical staff of the ward was supposed to conduct all the necessary clinical and pathological examinations which could not be conducted immediately at the bedside. In spite of the mistake in appellation, this was the ward "clinical laboratory." It served a very useful purpose in its day, and even now many institutions have nothing better than this simple little spot set apart in the ward and consecrated to the examination of specimens which must be immediately, or almost immediately, investigated. In its time it was all that was required. When a specimen of urine, for instance, was to be examined, the medical attendant was satisfied to test it for certain simple abnormalities —sugar, albumen, derived acids, indican, casts and crystals, and blood or bile. For that purpose all that was necessary was a small spirit-lamp, a few test-tubes, a few simple reagents, and a simple microscope of relatively low power. Similarly the same armamentarium served for the examination of sputum. Later on, when blood examinations became a matter of routine, this armamentarium was increased by the addition of a blood-counting slide, a hæmoglobinometer, and a compound microscope. Still later it was found necessary further to increase the outfit by providing the means for testing other clinical specimens—chief among which may be mentioned fæces and cerebro-spinal fluid—in order to take advantage of the most recent aids to clinical diagnosis derived from the examination of body fluids and excreta. Gradually the old window-sill or cupboard no longer served for the complicated ritual that had to be gone through in order to get the best results—and only the best could serve. This has been the cause of the greater elaboration of the modern ward laboratory.

### The Necessity for the Ward Laboratory.

The advances in modern medicine made during the last twenty-five years have in some ways materially changed the methods of diagnosis of disease which were in vogue before that period. Formerly the diagnosis was mainly established on probabilities—probabilities which wide clinical and pathological experience had proved to be very sound, but still probabilities. To-day in most cases the diagnosis has to be made on certainties. The physician must justify it not by experience alone, but by his opinion based on facts derived from the careful, methodical, and detailed examination of body fluids and excreta. It is this, largely, that has made the provision of a ward laboratory indispensable in the modern hospital.

### A Practical Instance.

We may make the position clear by taking one or two common everyday cases by way of illustration. A patient is admitted suffering from enlarged glands. Formerly that diagnosis would have sufficed. Nowadays it is not a diagnosis; the glands are merely a symptom that has to be taken into account. The diagnosis is adenitis, which may be due to one of various causes—to tuberculosis, to sepsis, to syphilis, to malignant disease, to malaria or even sleeping sickness, or to blood disease. In order to find out which of these causes underlies the condition the physician has not only to examine the patient carefully, to find out his previous history and antecedental circumstances, but he has also in addition to investigate carefully the patient's blood, urine, saliva, sputum, fæces and cerebro-spinal fluid. It may be that the investigation is a very complicated one, involving numerous intricate chemical analyses, or equally time-taking bacteriological culture experiments. In such a case the examination is more properly conducted in the institutional laboratory, where every possible means of furthering the inquiry is at hand. But very often all that is required to give certainty, and therefore to clinch the diagnosis already tentatively made in the case by the careful consideration of probabilities, is to make a short examination at the bedside and to investigate the fluid or excreta in the ward laboratory.

In the case cited a sample of blood is drawn from the patient's ear; it is at once spread on a slide, stained, fixed, and placed under the microscope. Examination reveals the fact that the condition of the blood is greatly altered; the percentage of white cells is far in excess of the normal, and the character of these cells is abnormal. There is no doubt that the case is one of a blood disorder. By this initial test, carried on in the ward laboratory, the field of probability is much limited, and there is at least one definite certainty to go upon. Similarly if the examination of the blood reveals the fact that the organism of malaria or sleeping sickness is present, the diagnosis of either of these two diseases is clinched. Nor is it only in matters of diagnosis that the ward laboratory is indispensable. Daily examinations have to be made in many cases to find out how a patient may be progressing, how he is responding or not responding to treatment. Thus in the case of a diabetic patient it is necessary to make a daily examination of the urine to estimate the amount of sugar excreted, and further to find out, by estimating the percentage of certain other abnormal constituents, such as acetone and diacetic acid, how far the patient is progressing either way.

* Previous articles have appeared on Oct. 7, 14, 21, 28, Nov. 4, 18, Dec. 9, 30, Feb. 10, March 16, April 6 and 20.

